# Supplementary material for: Learning curve and surgical outcome of robotic assisted colorectal surgery with ERAS program
Source: Sci Rep. 2022 Nov 29;12:20566. doi: 10.1038/s41598-022-24665-w (PMC9709162; doi:10.1038/s41598-022-24665-w)
Supplement: Supplementary file 1 — Supplementary Information 1. [file 41598_2022_24665_MOESM1_ESM.zip › LSC Raw data-Ñ[▒K/127/N_ñw╝╨░O▒Kñσ.pdf]

W85 / W85

臺中榮民總醫院
手術室護理記錄

[redacted]

基本資料

- 室別： 開刀房-18室，手術科別： CRS+GS，手術類別： 急症，日期： 2021/06/05
- 進入時間： 1405 ； 麻醉開始時間： 1410 ； Time Out時間： 1448 ； 劃刀時間： 1449 ； 手術結束時間： 1750 送出時間： 1810 ；
- 麻醉方式： GE
  診斷：A-colon diverticulysis with abscess formation
  手術名稱：1.Laparoscopic right colectomy and anastomosis
  2.Laparoscopic
  手術局部用藥：NIL
  術前預防性抗生素： 無
- 手術醫師： 林俊余，陳維信，陳咨言，楊曙榮，陳璿夫
- 麻醉醫師： VS 沈靜慧，麻醉醫師： R 蔡仁富
-
  - 刷手護士A ： 陳妍希，時間： 1405，接替刷手一： 張秀敏，時間： 1500
  - 流動護士A ： 林湘芸，時間： 1405，接替流動一： 邱任宏，時間： 1500

皮膚狀況

A-V Shunt： 無
術前： 完整
記錄者： 上次簽章: 邱任宏
術後： 完整
記錄者： 上次簽章: 邱任宏

手術準備

手術臥式 單一手術臥式

-
  - 手術臥式： 仰臥
  - 使用附件： 矽膠枕頭
  - 皮膚準備： 10%Tr.Betadine，10%Aq Betadine，Hibiscrub
  - 電燒： 使用電燒: 電極片位置： 右大腿，記錄者： 上次簽章: 邱任宏

止血帶T： 無

植入物

- 植入物名稱/號碼/數量/部位：Ethicon Endo GIA 60-3.6*3/A-colon

，記錄者： 上次簽章: 邱任宏

標本送檢

- 細菌培養 無
- 細胞學 無
- 冰凍切片 無
- 病理標本
  組織名稱一：A-colon
  ,
  組織名稱二：IC Root
- 截肢 無

標本交付 標本： 2 袋，標本： 0 盒，標本： 0 瓶，細胞學： 0 管，Culture Tube： 0 管，被交付醫師： R 陳璿夫，

- 記錄者： 上次簽章: 邱任宏
- 時間： 1730

傷口術後敷料

傷口敷料： 傷口敷料： 無

- 請於圖上標示部位：

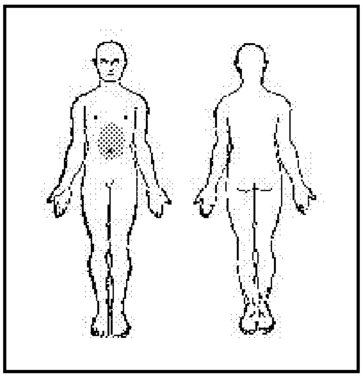

- 傷口敷料： 軟性敷料

導管

導管	天數	導管資訊	插入日期	插入時間	更換日期	拔除日期	拔除時間	拔除原因	預計拔除/更換	預計拔除/更換日期
Penrose Drain(1)	1	號碼:12 mm.; 部位:骨盆腔;	2021/06/05	1715						
Foley(1)	1	號碼:16 Fr.; 部位:膀胱;	2021/06/05	1435						

用物計數

- 手術中出血量： 少 c.c.
- 手術中尿量： 300 c.c.
-
    - 其它： 無

用物計數： 單一切口

- 1.器械： 正確，2.縫針： 正確，3.刀片： 正確，4.紗布： 正確，5.腹部墊： 未使用，6.紗球： 未使用，7.PEANUTS ： 未使用，8.紗條： 未使用，9.絲帶： 未使用，10.針頭： 未使用，11.Vessel loop： 未使用，12.Boots： 未使用，13.Jelco： 未使用，14.棉條： 未使用，
    - 第一次：
        - 刷手護士 ： 張秀敏
        - 流動護士： 上次簽章: 邱任宏
        - 時間： 1710

  ,
    - 第二次：
        - 刷手護士 ： 張秀敏
        - 流動護士： 上次簽章: 邱任宏
        - 時間： 1730

  ,
    - 第三次：
        - 刷手護士 ： 張秀敏
        - 流動護士： 上次簽章: 邱任宏
        - 時間： 1745

備註

備註： GS 5mm Scope
CRS 腹腔鏡 NO.1

- 記錄者： 上次簽章: 邱任宏
- 時間： 1500

填寫護理人員 邱任宏

正式摘要

W85 / W85

doctorRank:B
